# Improvement of Nanostructured Polythiophene Film Uniformity Using a Cruciform Electrode and Substrate Rotation in Atmospheric Pressure Plasma Polymerization

**DOI:** 10.3390/nano12010032

**Published:** 2021-12-23

**Authors:** Jae Young Kim, Hyo Jun Jang, Gyu Tae Bae, Choon-Sang Park, Eun Young Jung, Heung-Sik Tae

**Affiliations:** 1 School of Electronic and Electrical Engineering, College of IT Engineering, Kyungpook National University, Daegu 41566, Korea; jyk@knu.ac.kr (J.Y.K.); bs00201@knu.ac.kr (H.J.J.); doctor047@knu.ac.kr (G.T.B.); 2 Department of Electrical and Computer Engineering, College of Engineering, Kansas State University, Manhattan, KS 66506, USA; purplepcs@ksu.edu; 3 School of Electronics Engineering, College of IT Engineering, Kyungpook National University, Daegu 41566, Korea

**Keywords:** atmospheric pressure plasma polymerization, conjugated polymer film, plasma polymerization, plasma synthesis, polythiophene nanostructure

## Abstract

In atmospheric pressure (AP) plasma polymerization, increasing the effective volume of the plasma medium by expanding the plasma-generating region within the plasma reactor is considered a simple method to create regular and uniform polymer films. Here, we propose a newly designed AP plasma reactor with a cruciform wire electrode that can expand the discharge volume. Based on the plasma vessel configuration, which consists of a wide tube and a substrate stand, two tungsten wires crossed at 90 degrees are used as a common powered electrode in consideration of two-dimensional spatial expansion. In the wire electrode, which is partially covered by a glass capillary, discharge occurs at the boundary where the capillary terminates, so that the discharge region is divided into fourths along the cruciform electrode and the discharge volume can successfully expand. It is confirmed that although a discharge imbalance in the four regions of the AP plasma reactor can adversely affect the uniformity of the polymerized, nanostructured polymer film, rotating the substrate using a turntable can significantly improve the film uniformity. With this AP plasma reactor, nanostructured polythiophene (PTh) films are synthesized and the morphology and chemical properties of the PTh nanostructure, as well as the PTh-film uniformity and electrical properties, are investigated in detail.

## 1. Introduction

Atmospheric pressure (AP) plasma polymerization is a synthesis method in which vaporized monomer molecules successively interact with charged particles and excited species in nonthermal plasma at AP to generate various radicals and reactive species, react with each other, and form various polymers [1,2,3,4,5,6,7]. In nonthermal AP plasma, which is a partially ionized gas containing a large number of reactive, neutral, and charged particles, the plasma energy is mainly directed toward electrons with high mobility, and the ions and neutral species in the plasma medium remain at room temperature [8,9,10,11,12,13,14]. Thus, in nonthermal AP plasma, diverse reactive species and high-energy electrons exist at low gaseous temperatures, and this nonthermal behavior has great potential for applications in synthesizing thermally sensitive materials, including polymers. AP plasma polymerization is a convenient and effective approach for depositing conjugated nanostructured polymer films, because this process has the great advantage of not requiring a vacuum chamber and the relevant vacuum equipment for plasma polymerization [15,16,17,18].

In our previous reports, the device configuration of a guided wide tube and a substrate stand have been proposed to effectively block ambient air during AP plasma polymerization and to make the reactive monomer species stay longer inside the plasma reactor [19,20,21,22]. By confining the plasma medium using the guided tube and the substrate stand, the spatial uniformity of the discharge gas and the monomer vapor could be ensured for a longer period of time, enabling the intensive deposition of nanostructured polymeric films onto the substrate. Our research group recently reported on low-voltage-driven AP plasma polymerization using a bare electrode, in which the electrode was exposed to the plasma medium [23,24]. The AP plasma reactor with a pin-type bare electrode did not require charging and discharging of the capacitive dielectric barrier and, thus, the AP plasma could be generated at a lower voltage. This advantage also made it possible to use higher amounts of monomers in the polymerization process even during low-power plasma operation. However, the drawback of this AP plasma reactor is that it has a structural limitation in securing a uniform and wide polymerization processing area in spite of structural optimization efforts, because it uses a single pin-shape electrode. In order to apply the polymer film deposited by the AP plasma polymerization method to various research and industrial fields, it is important to ensure the film uniformity of the synthesized polymer films. Therefore, it is necessary to develop a new AP plasma reactor that can more easily expand the processing area in two dimensions.

In this study, we propose a new AP plasma reactor with a cruciform (cross-shaped)-bare-electrode structure in which two bare wires are crossed laterally in two dimensions, and optimize the experimental conditions to obtain uniform and wide nanostructured polythiophene (PTh) films in the range of several square centimeters. In particular, AP plasma reactors are fabricated with different lengths of tungsten wire exposed to the plasma space, and the discharge characteristics and PTh-film uniformity are examined with respect to the bare length of the electrode. In addition, AP plasma polymerization is performed while rotating the substrate stand using a turntable in order to obtain a uniformly nanostructured PTh film.

## 2. Materials and Methods

### 2.1. Experimental Setup for AP Plasma Polymerzation

**Figure 1:**
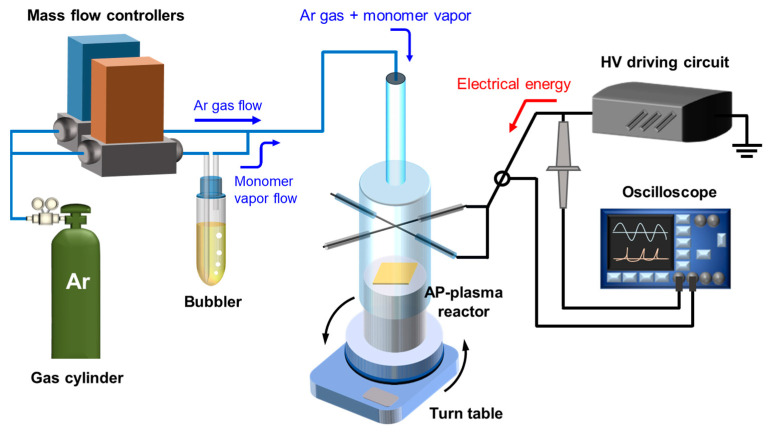
Schematic diagram of the AP plasma polymerization system combined with gas supply, AP plasma reactor, and high-voltage power supply.

A schematic diagram of the AP plasma polymerization system employed in this study is presented in Figure 1. The gas feedline was divided into two units for independent control of the gas flow rates for AP plasma generation and vaporized monomer injection. Argon (Ar) gas (HP grade with a purity of 99.999%) was used as the discharge gas at a flow rate of 1000 standard cubic centimeters per minute (sccm). Liquid thiophene monomer with a molecular weight of 84.14 g∙mol^−1^ (Sigma-Aldrich Co., St. Louis, MO, USA) was vaporized using a glass bubbler supplying an Ar flow rate of 100 sccm. Using an inverter-type driving circuit, a sinusoidal high voltage with a peak value of 10 kV and a frequency of 28 kHz was applied to the powered electrode of the AP plasma reactor. A turntable (T10, Excam, Busan, Korea) was used to rotate the substrate stand of the AP plasma reactor.

To monitor the electrical characteristics of the generated plasma, the voltage and current waveforms from the powered electrode were displayed on a digital oscilloscope (TDS 3014B, Tektronix Inc., Beaverton, OR, USA) using a high-voltage probe (P6015A, Tektronix Inc., Beaverton, OR, USA) and a current transformer (4100, Pearson Electronics Inc., Palo Alto, CA, USA). 

Photographs of the AP plasma reactor and the glass disk substrates were obtained using a digital single-lens reflex camera (D5300, Nikon Corp., Tokyo, Japan) equipped with a Macro 1:1 lens (Tamron SP AF 90 mm F2.8 Di, Tamron Co., Ltd., Saitama, Japan), and an intensified charge-coupled device (ICCD) camera (PI-MAX II, Princeton Instruments Inc., Trenton, NJ, USA) was used in shutter mode to identify the spatial distribution of the generated glow plasma.

For iodine (I_2_) doping, nanostructured PTh films deposited onto gold interdigitated electrode (IDE)-patterned silicon (Si) substrates were placed in a container with 2 g of solid I_2_ pellets (Sigma-Aldrich Co., St. Louis, MO, USA, 99.99%) and vacuum sealed for 30 min using a vacuum sealer packaging machine (VP-5700, Intropack, Pyeongtaek, Korea). The IDE had an interdigitated comb-shaped two-electrode structure with 20 pairs of microelectrodes. The width of the IDE was 10.8 µm, and the spacing between the adjacent IDEs was 2.54 µm.

### 2.2. Analysis and Characterization of the Nanostructured Polythiophene Film

The surface and cross-sectional morphology, thickness, and vertical orientation of the nanostructured PTh films on Si substrates were observed using field emission scanning electron microscopy (FE-SEM; SU8220, Hitachi High-Technologies, Tokyo, Japan) with accelerated electrons at a voltage of 3 kV and a current of 10 mA. The samples were coated with platinum to avoid surface-charging problems during analysis.

The thickness profile of the nanostructured PTh film was measured at 15 positions with an interval of 2 mm using a stylus profiler (P-7, KLA Tencor Corp., Milpitas, CA, USA) at the Korea Basic Science Institute (KBSI, Busan, Korea). Measurements were performed while moving the stylus in contact with the PTh-film surface at a scan speed of 200 μm∙s^−1^.

The chemical composition of the PTh films synthesized under different conditions were compared via Fourier-transform infrared spectroscopy (FTIR; Vertex 70, Bruker, Ettlingen, Germany) at KBSI (Daegu, Korea). The attenuated total reflection (ATR) FTIR spectra were measured by averaging 128 scans in the range of 650 to 4000 cm^−1^ at a wavenumber resolution of 0.6 cm^−1^.

High-resolution transmission electron microscopy images were taken with a Titan G2 ChemiSTEM Cs Probe (FEI Company, Hillsboro, OR, USA) transmission electron microscope operating at 200 kV. A transmission electron microscopy (TEM) sample of PTh nanoparticles was prepared by depositing a 10-µL solution (ultrasonically dispersed in DI water) onto carbon-coated copper grids and drying in air.

The X-ray photoelectron spectroscopy (XPS; ESCALAB 250XI, Thermo Fisher Scientific, Waltham, MA, USA) was carried out in order to investigate the surface chemical compositions, using monochromatic Al Kα X-ray source (hυ = 1486.71 eV) operated at 15 kV and 20 mA. The pressure in the analyzing chamber was maintained at 10^−8^ Pa or lower during analysis and the size of the analyzed area was 500 µm × 500 µm. Spectra were acquired with the angle between the direction of the emitted photoelectrons and the surface equal to 60°. The estimated analyzing depth of the used XPS setup was 8 to 10 nm. The high-resolution spectra were taken in the constant-analyzer-energy mode with 200 eV for the survey scan and a 50 eV pass energy for the element scan. The value of 285.5 eV of the C 1s core level was used for the calibration of the energy scale. To curve fit the high-resolution C 1s, S 2p, and O 1s peaks, the deconvolution of the C 1s, S 2p, and O 1s peaks was analyzed by the Thermo Advantage software. The peaks were deconvoluted using Gaussian–Lorentzian peak shapes (constrained between 80% and 100% Gaussian) and the full-width at half maximum (FWHM) of each line shape was constrained between 2.0 and 3.0 eV.

## 3. Results and Discussion

### 3.1. Newly Designed AP Plasma Reactor with Cruciform Bare Electrode

**Figure 2:**
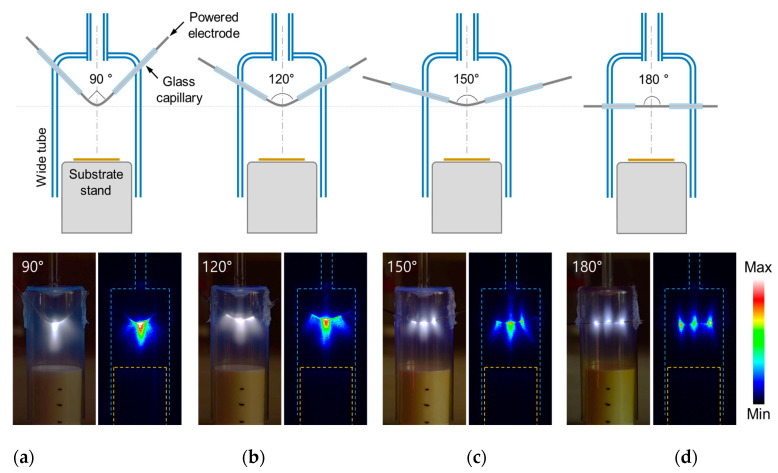
Optical observation (digital photographs and ICCD images) of glow plasma according to bending angle of tungsten wire electrode: (**a**) 90 degrees, (**b**) 120 degrees, (**c**) 150 degrees, and (**d**) 180 degrees.

Unlike a pin-type bare electrode structure in which the electric field is locally concentrated at the end of the pin-type electrode due to its protruding shape [23,24,25], it is necessary to first check where the discharge occurs in a plasma reactor with a powered electrode in which the wire is positioned parallel to the substrate. The discharge behaviors were tested by placing a bare wire in the center of the plasma reactor and varying the bending angle of the wire protruding toward the substrate as depicted in Figure 2. A 0.5 mm-diameter tungsten wire was used as the power electrode and covered with a glass capillary, and 20 mm was left exposed in the center of the wire for plasma generation.

As shown in Figure 2a, when the bare part of the wire was bent at 90 degrees, the discharge occurred only around the bending area similar to the discharge of a pin-type electrode. When the bare part of the electric wire was bent at 120 degrees, the discharge in the bending part at the center was weakened and the discharge spread to the entire bare electrode (Figure 2b). As the bending angle of the wire further increased to 150 and 180 degrees, the discharge at the central bending area gradually weakened, and two additional discharge regions were created at the boundaries of the bare wire and the glass capillary (Figure 2c,d). Therefore, a total of three discharge regions were formed inside the AP plasma reactor. As a result of this test, it was observed that the bending angle of the wire was very critical in determining the location and behavior of the discharge when the gas flow and operating voltage conditions were kept the same. In particular, when the bare part of the electric wire was not bent at all and, thus, there was no protruding part (Figure 2d), the discharge was strongly generated by localized electric-field enhancement at the two boundary regions between the bare wire and the glass capillary [26,27,28]. On the other hand, since the applied voltage did not change, the central discharge became weaker as the discharge intensity at the boundary increased.

To extend the experiments in Figure 2 to two dimensions, our research group developed an AP plasma reactor using two intersecting tungsten wires as a common power electrode. To designate the area where the discharge occurred in the AP plasma reactor, the intersection of the two wires was left as bare wire, and the rest of the electrode was covered with glass capillary tubes. The part where the two tungsten wires cross at 90 degrees was placed in the center of the wide tube and vertically aligned with the gas-emitting region. The detailed experimental conditions of this study are listed in Table 1. In addition, in this study, several parametric studies were performed in order to identify the optimal experimental conditions for depositing PTh films, which are summarized in Table 2. 

### 3.2. Glow Discharge Behaviors during AP Plasma Polymerization

**Figure 3:**
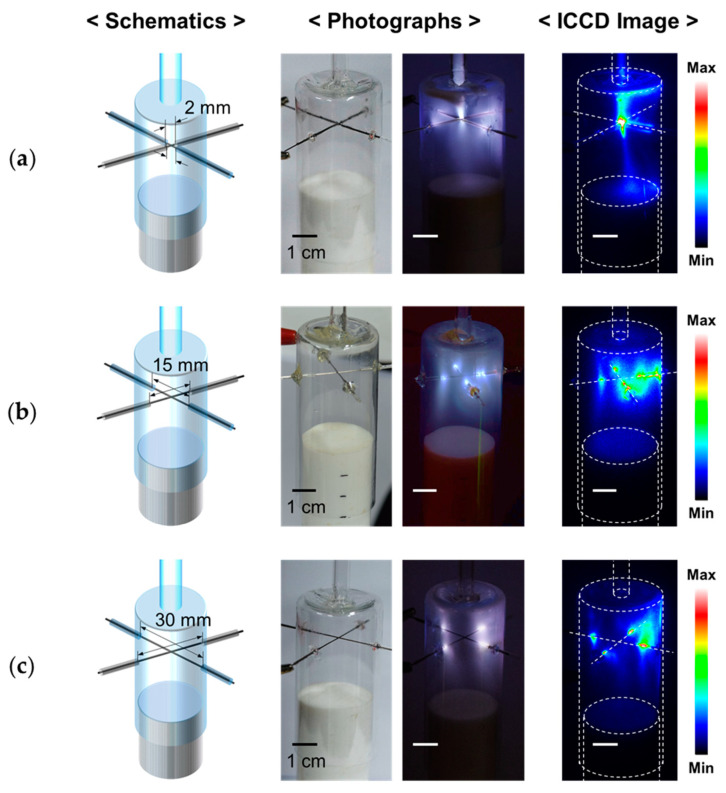
Optical observation of glow plasma according to length of the bare part of the cruciform wire electrode: (**a**) 2 mm, (**b**) 15 mm, and (**c**) 30 mm.

Three AP plasma reactors with bare-wire lengths of 2 mm, 15 mm, and 30 mm were fabricated, and the discharge characteristics and polymer film uniformity were investigated according to the length of the bare electrode. In the cruciform wire electrode partially covered with the glass capillary, it was observed that the glow discharge occurred at the boundary between the bare-wire part and the capillary, based on the observation of the digital photographs and the ICCD images. When the length of the bare-wire part was very small (2 mm), AP plasma was generated only in the central bare part of the cruciform wire electrode as designed (Figure 3a). As the length of the bare-wire part increased to 15 mm and 30 mm, the boundary point where discharge occurred gradually moved away from the center, and the discharge region was divided into fourths along the cruciform wire electrode, thereby successfully expanding the discharge volume (Figure 3b,c).

As shown in Figure 3a–c, when the length of the bare-wire part was increased, the discharge initiation point shifted, but the discharge area was not widened in the AP plasma reactor. The AP plasma reactor of Figure 3a had an electrode structure in which the discharge was concentrated in the center, whereas in the AP plasma reactor of Figure 3b, four discharge-generation regions were appropriately and evenly distributed in the polymerization space. However, when the length of the bare wire was increased to 30 mm, it was observed that the four plasma regions were very close to the sidewall of the wide tube as shown in Figure 3c, and the region where strong polymerization occurred also moved toward the sidewall.

### 3.3. Observation of Uniformity of the PTh Film

To investigate the uniformity of the PTh-film deposition in the entire polymerization region inside the AP plasma reactor according to the length of the bare wire, PTh films were synthesized on circular substrates with diameters of 30 mm, which was the same size as the substrate stand, and the spatial distribution of the deposited PTh film was observed. To better observe the deposited PTh film, a transparent glass disk was used as the substrate and polymerization took place for only 5 min to prevent the films from being too thick in this test. As mentioned, glow plasmas successfully occurred at the four boundaries between the bare-wire parts and the covered glass capillaries in the AP plasma reactor, but it was difficult to make the four discharges exactly the same. Even if the experimental setup and conditions were carefully managed, the glow plasmas generated at the four boundaries could not be perfectly matched. This uneven plasma state caused the nonuniformity of the polymerized film.

**Figure 4:**
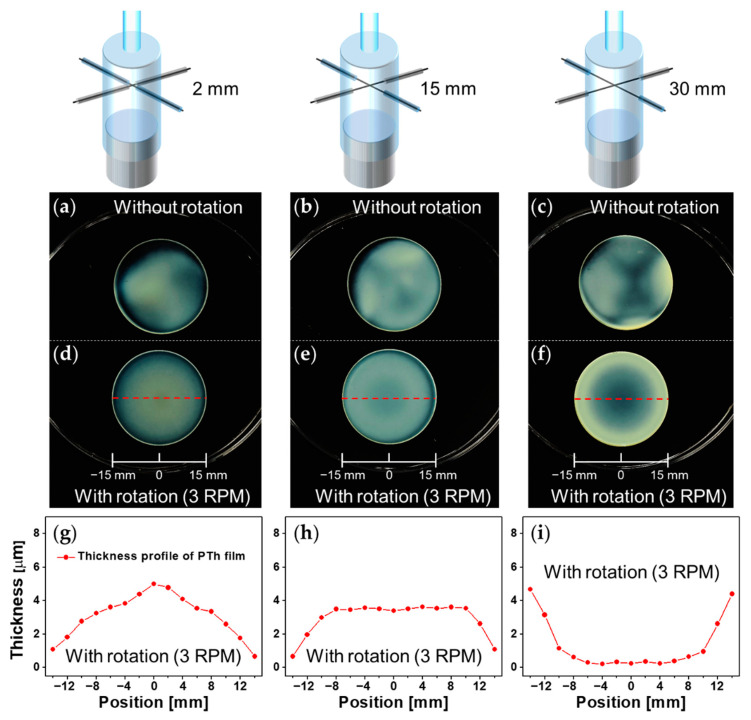
(**a**–**c**) Photographs of PTh films deposited onto glass disks with bare-wire lengths of (**a**) 2 mm, (**b**) 15 mm, and (**c**) 30 mm in an AP plasma reactor without substrate rotation; (**d**–**f**) Photographs of PTh films deposited onto glass disks with bare-wire lengths of (**d**) 2 mm, (**e**) 15 mm and (**f**) 30 mm with substrate rotating at 3 rpm; (**g**–**i**) Thickness profiles of the rotated nanostructured PTh films in (**d**–**f**).

As shown in Figure 4a–c, the uniformity of the PTh film along the length of the bare-wire part was investigated. In all three cases, it was observed that the resulting PTh films could not be deposited completely uniformly in the entire polymerization region. When the length of the bare-wire part was 2 mm, the PTh film was mainly deposited near the center of the glass disk along the generated plasma shape, and thus was not uniform as a whole region (Figure 4a). When the length of the bare-wire part increased to 15 mm, the PTh film that was deposited onto the glass disk showed an uneven pattern along the cruciform electrode due to the nonuniform plasma state (Figure 4b). In particular, when the length of the bare part was 30 mm, the deposition regions of the PTh film did not overlap each other because the glow plasmas that were generated in the four regions were far apart from each other, resulting in a very nonuniform film (Figure 4c).

### 3.4. Substrate Rotation for Improvement of Uniformity of the PTh Film

To resolve this issue of the nonuniformity of the PTh film, the substrate stand was rotated with a turntable during the polymerization process. The substrate stand was rotated very slowly at three revolutions per minute (3 RPM) to secure the PTh-film uniformity in the azimuthal direction and to minimize the influence that it may have on the substrate by rotation, such as centrifugal force. Therefore, the PTh films were deposited onto glass disks with the substrate rotating 15 times (5 min × 3 RPM) as shown in Figure 4d–f. Figure 4g–i show the thickness profiles of the rotated PTh films that were measured using a stylus profiler at 15 positions with an interval of 2 mm along the red dashed lines on the PTh films in Figure 4d–f. As a result, the most uniform PTh film was obtained when the length of the cruciform bare wire was 15 mm and the substrate stand was rotated. For this reason, the morphological and chemical properties of the PTh films, which will be discussed in the next sections, were examined using this AP plasma reactor with a bare-wire length of 15 mm and a rotating substrate stand.

### 3.5. Morphological and Chemical Properties of the Nanostructured PTh Film

**Figure 5:**
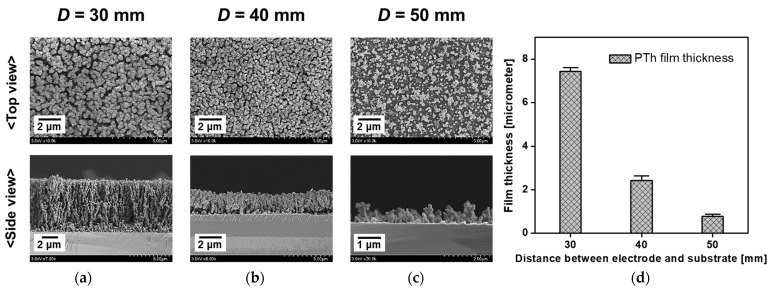
FE-SEM images of nanostructured PTh films deposited onto silicon substrates when the distances between the electrode and the substrate (*D*) were (**a**) 30 mm, (**b**) 40 mm, and (**c**) 50 mm and (**d**) comparison of thickness of PTh films.

Because the cruciform powered electrode was placed parallel to the substrate in the proposed AP plasma reactor, determining the spacing between the electrode and the substrate was very critical to the growth of the nanostructured PTh film. As shown in Figure 5a–c, the FE-SEM images depict the surface morphology and film thickness of the nanostructured PTh film that was deposited onto the Si substrate according to the distance between the substrate and the electrode. When the distance between the electrode and the substrate (*D*) was 30 mm, a longitudinally oriented PTh nanostructure was deposited onto the Si substrate with a thickness of 7.2 μm, despite the short polymerization time of 10 min (Figure 5a,d). In general, when the polymerization reaction takes place in the discharge space and the synthesized material falls onto the substrate, it is difficult to observe vertically aligned nanostructures in the prepared polymer film. The growth of vertically aligned PTh nanostructures as shown in Figure 5a indicated that the chemical bonding proceeded on the substrate. Therefore, the prepared nanostructured PTh film can be expected to have good adhesion to the substrate. In this regard, our research group recently examined the adhesion properties of PTh films synthesized by the AP plasma reactor [22]. As a result, the PTh films showed excellent adhesion properties on glass, Si, and PET substrates. On the other hand, as D increased, the film thickness decreased significantly (Figure 5b–d). In particular, when D was increased to 50 mm, the surface morphology of the PTh nanostructures showed a low density and their thickness was observed to be less than 1 μm. 

**Figure 6:**
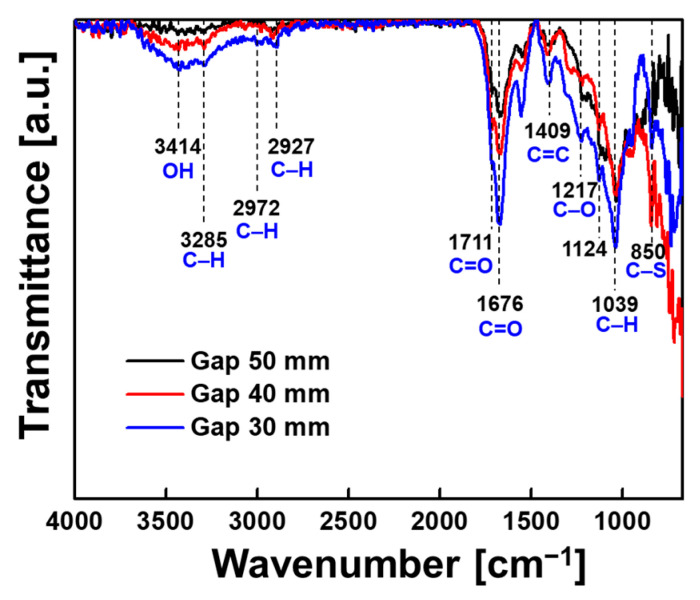
Comparison of ATR-FTIR spectra of deposited nanostructured PTh films when the distance between the electrode and the substrate of the plasma reactor was changed to 30 mm, 40 mm, and 50 mm.

Figure 6 depicts the ATR-FTIR spectra of the PTh films, which show the characteristic peaks of PTh films as follows: C–S bending (850 cm^−1^), C–H in-plane deformation (1039 cm^−1^), C–O stretch bending (1217 cm^−1^), C=C asymmetric stretching vibration mode of the thiophene ring (1411 cm^−1^), C=O symmetric stretching vibration modes of the thiophenering (1675 cm^−1^ and 1711 cm^−1^), and C–H stretching vibrations (2931 cm^−1^, 2977 cm^−1^, and 3282 cm^−1^) [29,30,31]. When *D* was 30 mm, it was observed that the intensity of most of the main peaks increased in the region of greater than 1039 cm^−1^ on ATR-FTIR, indicating that the contribution of the main chemical groups constituting PTh was improved. Although the PTh films were successfully deposited onto the substrate under all *D* conditions, the quality of the PTh films changed as *D* was varied. Consequently, we tentatively determined the optimal *D* to be 30 mm based on the results shown in Figure 5 and Figure 6.

**Figure 7:**
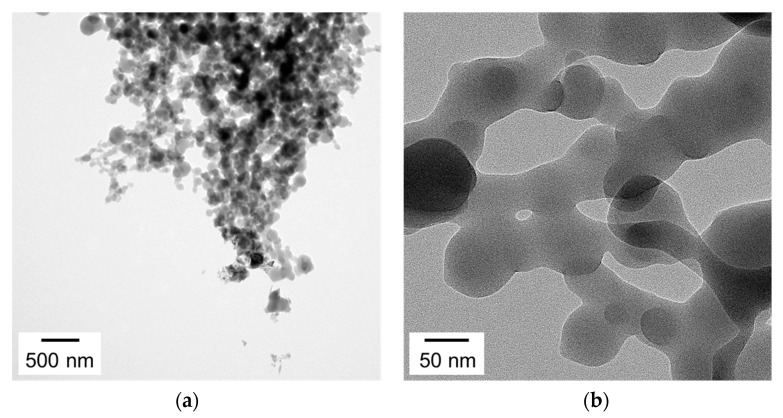
Transmission electron microscopy (TEM) images of PTh nanoparticles prepared via the proposed AP plasma reactor with (**a**) low magnification and (**b**) high magnification.

Figure 7 shows the TEM images of the PTh nanoparticles generated by the proposed AP plasma reactor. The TEM sample of the PTh nanoparticle cluster was collected from the nanostructured PTh film that was deposited onto the silicon substrate when *D* was 30 mm (Figure 7a). It was observed that PTh nanoparticles with diameters of approximately 20–60 nm were interconnected to form many cross-linked networks (Figure 7b), indicating that PTh nanoparticles could be effectively synthesized.

**Figure 8:**
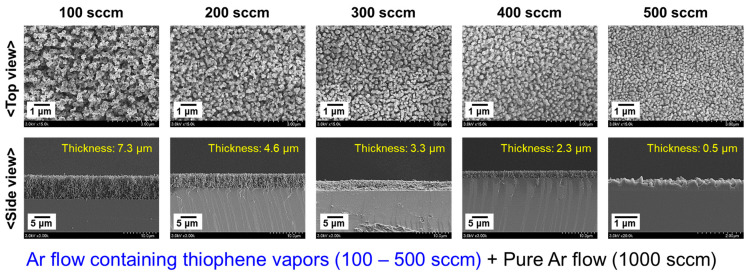
FE-SEM images of nanostructured PTh films deposited onto silicon substrates as Ar flow for vaporized thiophene increased from 100 to 500 sccm.

To determine the optimal amount of thiophene monomer under the experimental condition where *D* is 30 mm, the morphological properties of the deposited PTh film were investigated while changing the Ar flow applied to the bubbler containing liquid thiophene monomer. As the Ar flow for the vaporized thiophene increased from 100 to 500 sccm, the thickness of the nanostructured PTh films gradually decreased while the grain size of the PTh nanoparticles did not significantly change, as shown in Figure 8. When only the amount of vaporized thiophene molecules increased under the experimental conditions that were employed in this study, the Ar discharge became unstable, which adversely affected AP plasma polymerization and the growth rate of the PTh film. If the driving voltage of the plasma is increased, it is expected that the growth rate of the PTh film can be increased even at Ar flows for the thiophene vaporization of 200–300 sccm. However, when the driving voltage increased, the imbalance of the four discharge regions became more severe. Therefore, the Ar gas flow rate for the thiophene vapor injection was optimized to 100 sccm under the current experimental conditions. This is because a nanostructured PTh film with a sufficient thickness of 5 to 10 μm can be obtained with a short process time of 10 min while maintaining a low driving voltage.

**Figure 9:**
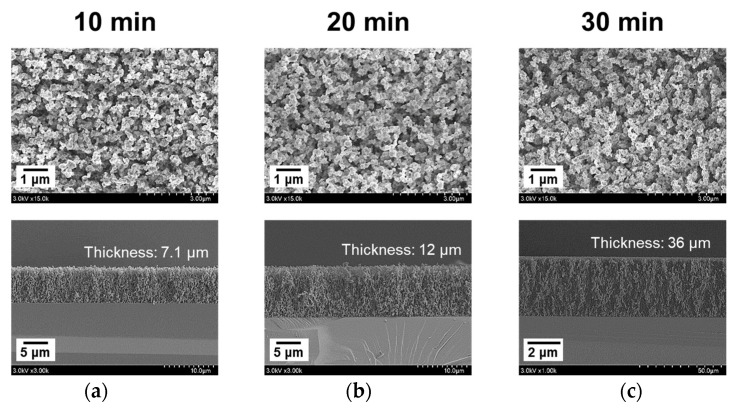
FE-SEM images of nanostructured PTh films as polymerization process time changed; (**a**) 10 min, (**b**) 20 min, and (**c**) 30 min.

At the optimal gas conditions that combined a pure Ar flow of 1000 sccm and an Ar flow containing monomer vapor of 100 sccm, AP plasma polymerization was tested with increasing process times from 10 to 30 min in order to check the thickness of the nanostructured PTh films deposited onto the Si substrates (Figure 9). Under the same experimental conditions, except for the polymerization time, the grain sizes of the PTh nanoparticles remained almost unchanged, and the thicknesses of the nanostructured PTh films increased as a function of time. As the PTh nanoparticles accumulated in the vertical direction, the vertically aligned nanostructures were densely formed, then bundles of these nanoparticles formed a film on the Si substrate. After polymerization for 30 min, a nanostructured PTh film of 35 μm or higher can be obtained, confirming that the proposed polymerization process is a fast synthesis process at room temperature and AP conditions.

### 3.6. Comparison of Nanostructured PTh Films Synthesized in the Plasma Remote and Coupling Modes

As shown in Figure 5, the optimal distance between the electrode and the substrate, *D*, was tentatively determined as 30 mm in this study. The case where *D* was less than 30 mm was examined in detail. The volume of the glow-plasma region was controlled by the volume of the plasma reactor, the presence of the counter electrode, the discharge gas (Ar) flow, the additives introduced into the plasma, and the applied voltage. By controlling the volume of the glow-plasma region and the distance between the electrode and the substrate, the generated plasma can be in contact with the substrate. In this experiment, when *D* was 20 mm, the glow plasma that was generated inside the guide tube may or may not have come into direct contact with the substrate due to the subtle and uncontrollable differences among the four discharges. That is probably due to the instability of the amount of vaporized thiophene monomer in this AP system. The case where the glow plasma that was generated in the discharge space contacts the substrate is called the plasma coupling mode, and the case where the plasma does not contact the substrate is called the plasma remote mode [32,33].

**Figure 10:**
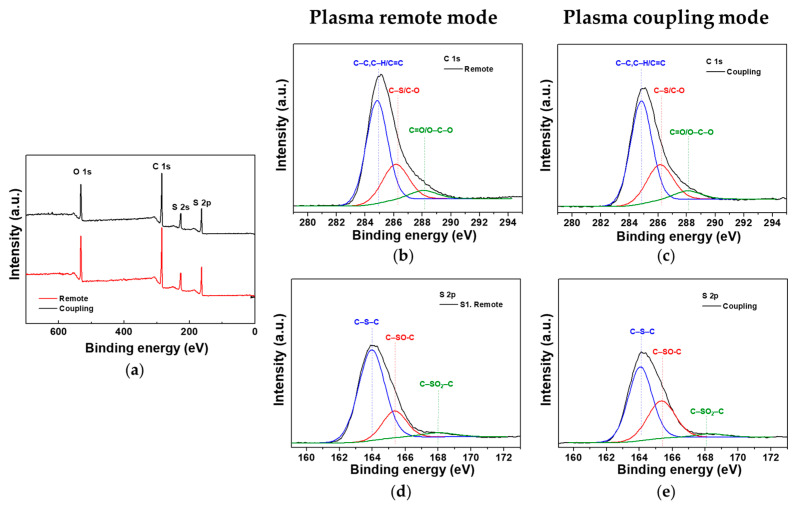
(**a**) XPS survey spectra of nanostructured PTh films synthesized under the plasma coupling and remote conditions. High-resolution XPS spectra of C 1s of PTh film prepared using the plasma (**b**) remote and (**c**) coupling modes. High-resolution XPS spectra of S 2p of PTh film prepared using the plasma (**d**) remote and (**e**) coupling modes.

Because this AP plasma reactor generated glow plasmas in four places simultaneously, it could have caused slight differences among the four discharges. In addition, the proposed cruciform bare electrode potentially had an issue of discharge instability because it was directly exposed to the plasma medium. Therefore, the generated plasma volume (and length) may have varied slightly due to the relatively unstable discharge, which made it impossible to completely control the two plasma modes at a *D* of 20 mm. The chemical composition of the surface of PTh films that were obtained under the plasma coupling and remote conditions was analyzed using XPS results as shown in Figure 10. The XPS signals of the PTh films included specific signals of O 1s (532.1 eV), C 1s (285.5 eV), and S 2p (164.0 eV); these atomic concentrations are summarized in Table 3. The stoichiometric ratio of carbon to sulfur (C/S) of PTh was expected to be 4:1, because thiophene molecules have a ring structure with 4 carbons and 1 sulfur. In both the plasma remote and coupling condition, C/S ratios of nanostructured PTh films were satisfactory at 4.1 and 4.0, respectively.

Figure 10b–e show the high-resolution XPS spectra of C 1s and S 2p. In Figure 10b,c, the C 1s peak consisted of three distinctive component peaks attributed to C–C, C=C, C–H bonds (284.9 eV), C–S, C–O bonds (286.2 eV), and C=O, O–C–O bonds (288.1 eV) [29,34]. The presence of C–O, C=O bonds in the high-resolution XPS of C 1s were also confirmed by the FT-IR results. In Figure 10d,e, the S 2p peak could be decomposed into a C–S–C bond (164.0 eV), C–SO–C bond (165.3 eV), and C–SO_2_–C bond (168.2 eV), which were revealed by the aromatic ring of PTh [34,35]. The ratios of the deconvoluted components of C 1s and S 2p are summarized in Table 4. The presence of sulfur oxides in the PTh nanostructure synthesized at AP indicated that oxygen in the atmosphere was partially excited by plasma energy. Ratios of sulfur oxides (C–SO–C and C–SO_2_–C) to sulfide (C–S–C) were 0.33 and 0.64 for the plasma remote and coupling modes, respectively, meaning that more excited oxygen was involved in the synthesis process in the plasma coupling mode than in the plasma remote mode. Therefore, it was confirmed that more plasma energy was applied to the substrate with the PTh film in the plasma coupling mode.

**Figure 11:**
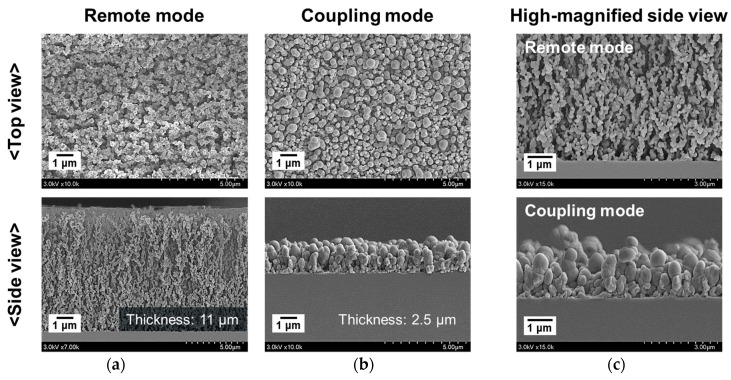
Comparison of FE-SEM images of plasma remote mode and coupling mode: (**a**) plasma remote mode, (**b**) plasma coupling mode, and (**c**) magnified side view FE-SEM images in the two different plasma modes.

As shown in Figure 11a,b, the morphology and thickness of the nanostructured PTh film was significantly different between the plasma remote mode and plasma coupling mode. In the plasma remote mode, the PTh nanostructure grew high in the vertical direction while maintaining small grain sizes, whereas in the plasma coupling mode the PTh nanostructure had large grain sizes but did not grow high in the vertical direction. As shown in the magnified side-view FE-SEM images of Figure 11c, when the PTh nanostructure was initially grown on a Si substrate in the plasma coupling mode, it formed small nanoparticles on the substrate. When the plasma energy was transferred directly to the substrate, the grain sizes of the PTh nanostructure grew. Thus, the grain sizes gradually increased from the substrate toward the PTh-film surface. Moreover, the granularity was relatively irregular as the PTh nanoparticles grew randomly from the substrate to the film surface in the plasma coupling mode. 

In the plasma remote mode, the plasma energy was primarily used to grow the nanostructured PTh film on the substrate by bonding the PTh nanoparticles in longitudinal alignment during the polymerization process time. Conversely, in the plasma coupling mode, the plasma energy was used to increase the grain size of the PTh nanoparticles. Therefore, the thicknesses of the resulting nanostructured PTh films differed by more than a factor of four due to the different plasma modes occurring under the same polymerization conditions. 

### 3.7. Electrical Properties of Nanostructured PTh Films Synthesized in the Plasma Remote and Coupling Modes

In order to use the fabricated nanostructured PTh film as an electrode or transducer for electronic devices, it is very important to provide electrical properties to the film. A widely used approach to making the conjugated polymers, including conductive PTh, is to dope a halogen element such as HCl or I_2_ as an electron acceptor [36,37,38]. An ex situ I_2_-doping process in which a doping procedure is performed after synthesizing a polymer film has been reported in many studies due to the advantages of the low-cost and simple process [39,40,41]. The I_2_-doping principle and characteristics of I_2_-doped conjugated polymer films have been reported in previous studies of our research group [33,42,43,44]. 

**Figure 12:**
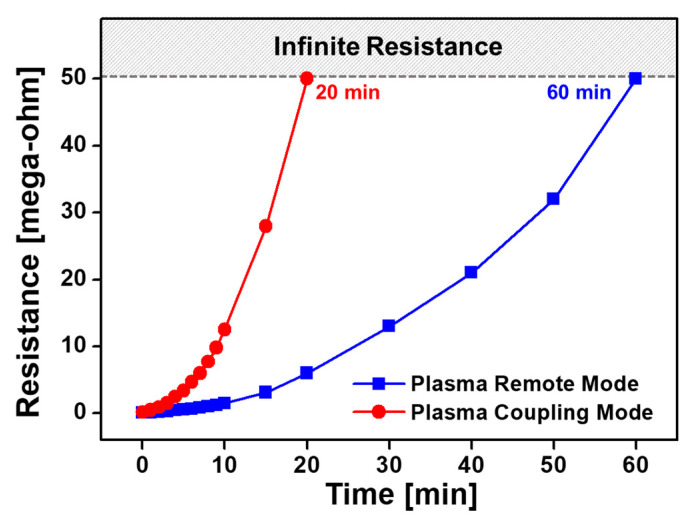
Changes in electrical resistance of iodine-doped PTh films on IDE substrates synthesized in plasma coupling and remote modes.

The electrical properties of conductive polymer materials are generally changed by water inside or on the surface of the nanostructure [44,45,46]. Thus, the resistance change of the I_2_-doped PTh film was measured according to the morphology of the nanostructured PTh film. Figure 12 shows the changes in the resistance of I_2_-doped PTh films synthesized in plasma coupling and remote modes with exposure time in ambient air. The measurement limit of the resistance of the I_2_-doped PTh film was 50 MΩ, and if it exceeded this limit then it was considered to have infinite resistance. In the plasma remote mode, the initial resistance of the I_2_-doped PTh film was measured to be 65 kΩ, and the resistance gradually increased in the atmosphere and reached the measurement limit, 50 MΩ, after 60 min. However, in the plasma coupling mode, the initial resistance was measured to be 150 kΩ and the resistance rapidly increased and reached the measurement limit in only 20 min. The dense and thick I_2_-doped PTh film that was obtained in the plasma remote mode was less affected by hydration under ambient air and had better stability to resistance changes. It was confirmed that the characteristics of the resistance change according to exposure time were in good agreement with the previously reported results [33]. The I_2_-doped PTh film with time-varying resistance properties can be expected to have a constant electrical conductivity when encapsulated with a sealing material to prevent the access of moisture and oxygen [42].

## 4. Conclusions

In this study, we proposed an AP plasma reactor with a cruciform electrode structure that had the advantage of an easy spatial expansion of the plasma medium, and investigated the morphological and chemical properties of the deposited PTh film using this plasm reactor. In the cruciform wire electrode partially covered with glass capillary tubes, the discharge occurred at the boundary where the covered capillary tubes ended. Thus, as the bare electrode part became longer, the boundary points where the discharge occurred gradually moved away from the center and the discharge region was divided into fourths in one AP plasma reactor. As a result, the glow-plasma medium expanded spatially. Even with careful management of the experimental setup and conditions, the plasma properties of the four boundaries cannot be completely matched, leading to the non-uniform deposition of the nanostructured PTh film. However, the uniformity of the nanostructured PTh film could be considerably improved by rotating the substrate stand using a turntable. The study of discharge characteristics and PTh-film properties according to the length of the bare electrode and the substrate rotation will be important experimental data for the development of advanced AP-plasma-polymerization methods that are capable of depositing a large-area polymer film using multiple electrodes. In addition, further studies on the characterization of AP glow plasmas that are generated from diverse cruciform or reticulated electrode structures will provide important clues to the infinite spatial scalability of polymer deposition under AP conditions.

## Figures and Tables

**Table 1:** Summary of experimental conditions for AP plasma polymerization.

Experimental Conditions		AP Plasma Reactor
Device Configuration	Powered electrode shape	Cruciform
	Electrode material	Tungsten wire
	Inner diameter of wide tube	34 mm
	Diameter of substrate stand	30 mm
	Distance between electrode and substrate	30 mm
Driving Conditions	Voltage waveform	Sinusoidal
	Plasma initiation voltage (V_p_)	4 kV
	Plasma driving voltage (V_p_)	10 kV
	Driving frequency	28 kHz
	Averaged power ^1^	4.40 W
Gas Conditions	Discharge and monomer carrier gas	Ar (HP grade: 99.999%)
	Gas flow rate for AP plasma discharge	1000 sccm
	Gas flow rate for thiophene monomer vapor	100 sccm
	Polymerization process time	10 min

^1^ Figure S1 provides waveforms of applied voltage, total current, power and discharge current.

**Table 2:** Summary of experimental conditions for parametric studies.

Parametric Studies					
Conditions	Variables				
Length of the bare-wire part of the electrode	2, 15, 30 mm	15 mm	15 mm	15 mm	15 mm
Distance between electrode and substrate	30 mm	30, 40, 50 mm	30 mm	30 mm	20 mm
Ar flow rate for AP plasma discharge	1000 sccm	1000 sccm	1000 sccm	1000 sccm	1000 sccm
Ar flow rate for thiophene monomer vapor	100 sccm	100 sccm	100–500 sccm	100 sccm	100 sccm
Polymerization process time	10 min	10 min	10 min	10, 20, 30 min	10 min
Results (Figures)	3 and 4	5 and 6	8	9	10–12

**Table 3:** Elemental concentrations of PTh films synthesized under the plasma coupling and remote conditions.

Conditions	C (%)	O (%)	S (%)	C/S
Plasma remote mode	65.4	18.5	16.1	4.1
Plasma coupling mode	66.6	17.0	16.5	4.0

**Table 4:** Peak assignments and percent composition of the C 1s and S 2p of PTh films prepared using the plasma remote and coupling modes.

Peak Assignment		Binding Energy (eV)	Composition (%)	
			Remote Mode	Coupling Mode
C 1s	C–C, C–H, C=C	284.9	62.6	65.3
	C–S, C–O	286.2	31.3	28.1
	C=O, O–C–O	288.1	6.1	6.6
S 2p	C–S–C ➀	164.0	75.2	60.8
	C–SO–C ➁	165.3	20.3	35.7
	C–SO_2_–C ➂	168.2	4.5	3.5
	(➁ + ➂)/➀	-	0.33	0.64

## Data Availability

Not applicable.

## Supplementary Materials

The following supporting information can be downloaded at: https://www.mdpi.com/article/10.3390/nano12010032/s1, Figure S1: Applied waveforms of (a) voltage, (b) total current, and (c) plasma discharge current under the conditions of Table 1.
