# Supplementary material for: Improvement of Nanostructured Polythiophene Film Uniformity Using a Cruciform Electrode and Substrate Rotation in Atmospheric Pressure Plasma Polymerization
Source: Nanomaterials (Basel). 2021 Dec 23;12(1):32. doi: 10.3390/nano12010032 (PMC8746814; doi:10.3390/nano12010032)
Supplement: Supplementary file 1 [file nanomaterials-12-00032-s001.zip › nanomaterials-1466996-supplementary.pdf]

Supplemental Materials

Improvement of Nanostructured Polythiophene Film Uniformity Using a Cruciform Electrode and Substrate Rotation in Atmospheric Pressure Plasma Polymerization

Jae Young Kim [1,†], Hyo Jun Jang [1,†], Gyu Tae Bae [1], Choon-Sang Park [2] Eun Young Jung [1,*], and Heung-Sik Tae [1,3,*]

1 School of Electronic and Electrical Engineering, College of IT Engineering, Kyungpook National University, Daegu 41566, Korea; jyk@knu.ac.kr (J.Y.K.); bs00201@knu.ac.kr (H.J.J.); doctor047@knu.ac.kr (G.T.B.)

2 Department of Electrical and Computer Engineering, College of Engineering, Kansas State University,
Manhattan, KS 66506, USA; purplepcs@ksu.edu

3 School of Electronics Engineering, College of IT Engineering, Kyungpook National University,
Daegu 41566, Korea

* Correspondence: eyjung@knu.ac.kr (E.Y.J.); hstae@ee.knu.ac.kr (H.-S.T.); Tel.: +82-53-950-6563 (H.-S.T.)

† These authors contributed equally to this work.

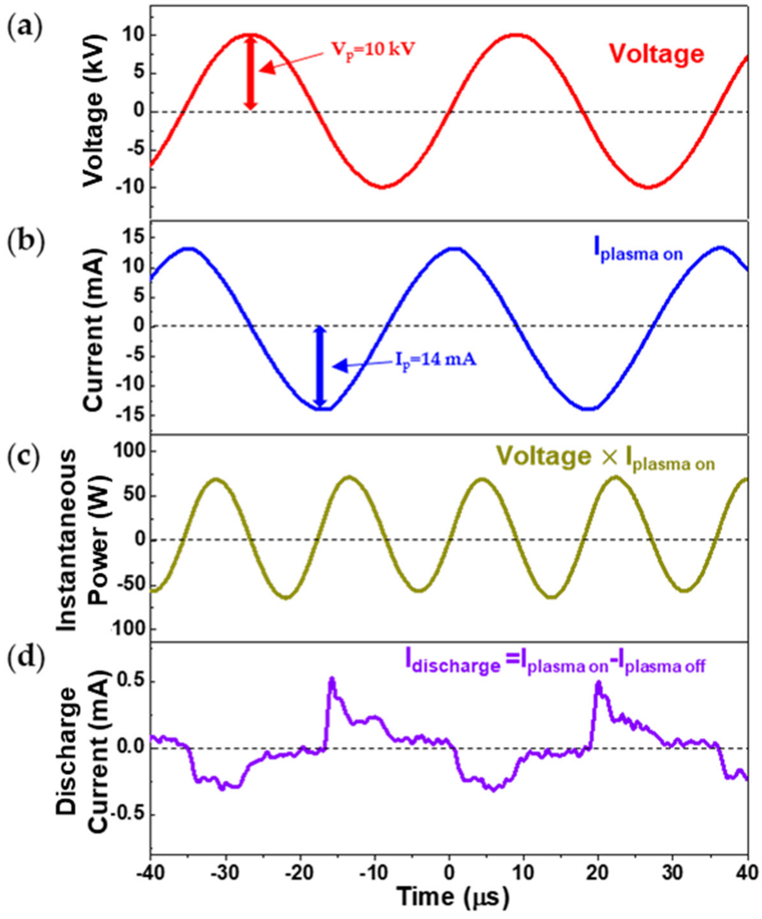

Figure S1. Applied waveforms of (**a**) voltage, (**b**) total current in plasma ON state, (**c**) instantaneous power, and (**d**) plasma discharge current under the conditions of Table 1.

The averaged power was calculated according to the following formula:

$$P = \frac{1}{T}\int_0^T U(t) \times I(t)dt$$

where T is the period of applied voltage, U(t) is the voltage signal, I(t) is the acquired current, and t is time, respectively. In Figure S1c, the integrated value of the waveform of the power during 1 period (from 0 μs to 35.6 μs) is 1.565×10^2 W·μs. Therefore, the averaged power during 1 period is 4.40 W.

In Figure S1d, the plasma discharge current is measured as the difference between the total current when the plasma is sustaining ($I_{total} = I_{discharge} + I_{displacement}$) and the total current when it is off ($I_{total} = I_{displacement}$).
